# Editorial: Microbiota and mitochondria: Impact on cell signaling, physiology, and disease

**DOI:** 10.3389/fmicb.2022.1056499

**Published:** 2022-10-18

**Authors:** Dionysios V. Chartoumpekis, Apostolos Zaravinos, Yiorgos Apidianakis, George Lagoumintzis

**Affiliations:** ^1^Service of Endocrinology, Diabetology and Metabolism, Lausanne University Hospital, University of Lausanne, Lausanne, Switzerland; ^2^Department of Life Sciences, School of Sciences, European University Cyprus, Nicosia, Cyprus; ^3^Cancer Genetics, Genomics and Systems Biology Laboratory, Basic and Translational Cancer Research Center (BTCRC), Nicosia, Cyprus; ^4^Department of Biological Sciences, University of Cyprus, Nicosia, Cyprus; ^5^Department of Pharmacy, School of Health Sciences, University of Patras, Patras, Greece

**Keywords:** microbiota, mitochondria, reactive oxygen species (ROS), cell-signaling, disease, microbial metabolites

The mitochondrion is an organelle of endosymbiotic origin that is central to the cell's energy production, contributing to cellular signaling and homeostasis. Higher eukaryotes have external symbionts, comprising their so-called microbiome, consisting primarily of bacteria found in various body surfaces, such as the mouth, skin, lungs and gut. Advances in the sequencing technology and the facile characterization of host bacteria at the species and even the gene level provide associations between microbiota profiles and diseases, including diabetes, obesity (Karlsson et al., 2013), neurodegenerative (Sarkar and Banerjee, 2019) and autoimmune diseases (Opazo et al., 2018). For instance, mitochondria exhibit reduced oxidative phosphorylation in the setting of diabetes and reduced plasticity in insulin-resistant subjects (Szendroedi et al., 2011). Also, in neurodegenerative diseases like Alzheimer's or Parkinson's, mitochondria show impaired bioenergetics (Knott et al., 2008).

Given that mitochondria likely evolved from ancient bacteria (Labbé et al., 2014), it is plausible that microbiota interact with the mitochondria of their host cells. A central factor may be reactive oxygen species (ROS), serving as a nexus of a microbiota-mitochondria crosstalk (Ballard and Towarnicki, 2020). However, the exact mechanisms of this communication remain unclear.

A PubMed search in September 2022 using the terms “microbiota and mitochondria” resulted in 382 papers (including 145 reviews), 80% of which were published within the last five years. This reflects that the precise high-throughput study of microbiota, mitochondria and their metabolites is only recently popularized.

Host mitochondria can affect the gut microbiome *via* ROS (Yardeni et al., 2019). Microbiota, in turn, can produce metabolites, such as short-chain fatty acids and secondary bile acids, which can alter the expression of genes, for example Pgc-1α, that regulate mitochondrial biogenesis and function (Clark and Mach, 2017). Hence, the crosstalk between microbiota and mitochondria is bidirectional and relatively hard to study, as it involves intimate host, microbe, and metabolite interactions (Figure 1).

**Figure 1:**
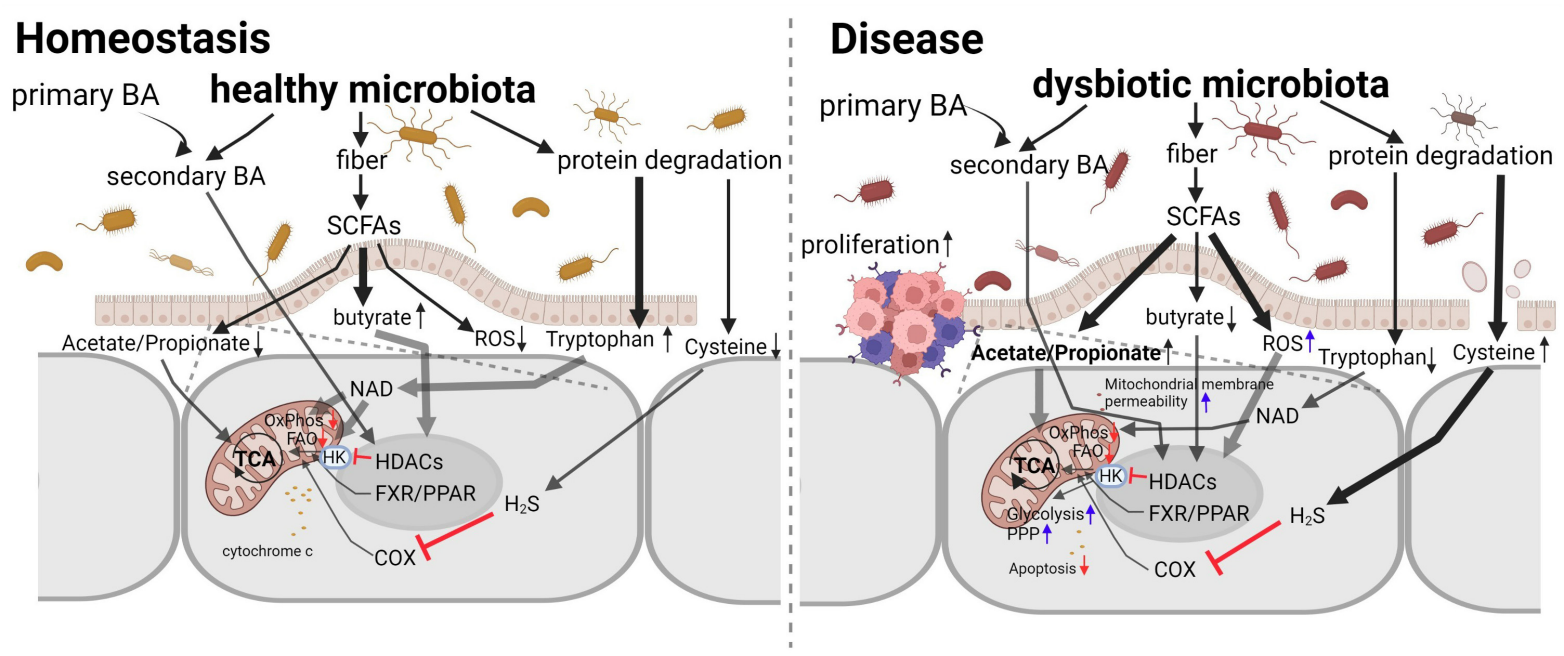
The interaction between microbiota and mitochondria in homeostasis and disease. Microbiota, both healthy and dysbiotic, produce factors that increase or decrease mitochondrial activity and intracellular ROS levels. Interventions targeting the microbiota to restore normal mitochondrial functions are promising novel therapeutic approaches for intestinal inflammation and cancer due to their plasticity in counteracting mitochondrial metabolic reprogramming. Adapted from Weber-Stiehl et al..

In this Research Topic (RT), we welcomed basic, translational, and clinical research studies on microbiota with emphasis on delineating the signal transduction pathways and crosstalk between microbiota and mitochondria and the effects of this interaction on physiology and related diseases. A pertinent paper (Weber-Stiehl et al.) published in this issue provides a very useful perspective on the interactions between intestinal microbiota and the mitochondria of enterocytes. There is evidence that intestinal microbiota metabolites and metabolic byproducts, such as short-chain fatty acids, butyrate, acetate and propionate, and secondary bile acids, as well as the amino acids, tryptophane and cysteine, facilitate a balanced mitochondrial function. Under intestinal inflammation, this interaction is altered, leading to a vicious cycle that perpetuates the inflammatory state and predisposes to cancer.

The impact of host metabolism is also illustrated in an interesting research paper describing differences in vaginal bacterial communities between estrus and non-estrus in giant pandas (Yue et al.). Specifically, species of the genera *Streptococcus, Escherichia*, and *Bacteroides* were significantly increased in the vagina of giant panda during estrus, providing a link between the reproductive hormonal state of estrus and vaginal bacterial composition. Further research may determine if carbohydrate and galactose metabolic pathways highly enriched in estrus pandas are related to the mitochondrial function (Aguer et al., 2011).

Along the same lines, this topic includes a translational research article describing distinct microbiota profiles in the placenta of pregnant women with premature rupture of membranes (PROM) or gestational diabetes mellitus (GDM) (La et al.). This study, along with others associating mitochondrial membrane damage with PROM (Fortunato and Menon, 2001) and mitochondrial dysfunction with GDM (Fisher et al., 2021), encourages further research on microbiota profiles associated with these pathologies and the role of mitochondrial function.

A deeper understanding of the interactions between mitochondria and microbiota is necessary to link microbial and host metabolism in health and disease mechanistically. Research similar to the works described in this RT may stimulate further investigation in this area and pave the way for biochemical studies focusing on the interplay between microbiota and mitochondria.

We would like to thank all the authors who contributed their original work to our RT and the reviewers for their valuable comments. We also thank the Frontiers Editorial Office for their support in providing us with the opportunity to host this RT.

## Author contributions

All authors have contributed substantially and equally to the article and approved the manuscript for publication.

## Conflict of interest

The authors declare that the research was conducted in the absence of any commercial or financial relationships that could be construed as a potential conflict of interest.

## Publisher's note

All claims expressed in this article are solely those of the authors and do not necessarily represent those of their affiliated organizations, or those of the publisher, the editors and the reviewers. Any product that may be evaluated in this article, or claim that may be made by its manufacturer, is not guaranteed or endorsed by the publisher.

## References

[B1] Aguer C. Gambarotta D. Mailloux R. J. Moffat C. Dent R. McPherson R.. (2011). Galactose enhances oxidative metabolism and reveals mitochondrial dysfunction in human primary muscle cells. PLoS ONE. 6, e28536. 10.1371/journal.pone.0028536 22194845 PMC3240634

[B2] Ballard J. W. O. Towarnicki S. G. (2020). Mitochondria, the gut microbiome and ROS. Cell Signal. 75, 109737. 10.1016/j.cellsig.2020.109737 32810578

[B3] Clark A. Mach N. (2017). The crosstalk between the gut microbiota and mitochondria during exercise. Front. Physiol. 8, 319. 10.3389/fphys.2017.00319 28579962 PMC5437217

[B4] Fisher J. J. Vanderpeet C. L. Bartho L. A. McKeating D. R. Cuffe J. S. M. Holland O. J.. (2021). Mitochondrial dysfunction in placental trophoblast cells experiencing gestational diabetes mellitus. J. Physiol. 599, 1291–1305. 10.1113/JP280593 33135816

[B5] Fortunato S. J. Menon R. (2001). Distinct molecular events suggest different pathways for preterm labor and premature rupture of membranes. Am. J. Obstet. Gynecol. 184, 1399–1405. discussion 1405-6. 10.1067/mob.2001.115122 11408859

[B6] Karlsson F. Tremaroli V. Nielsen J. Bäckhed F. (2013). Assessing the human gut microbiota in metabolic diseases. Diabetes. 62, 3341–3349. 10.2337/db13-0844 24065795 PMC3781439

[B7] Knott A. B. Perkins G. Schwarzenbacher R. Bossy-Wetzel E. (2008). Mitochondrial fragmentation in neurodegeneration. Nat. Rev. Neurosci. 9, 505–518. 10.1038/nrn2417 18568013 PMC2711514

[B8] Labbé K. Murley A. Nunnari J. (2014). Determinants and functions of mitochondrial behavior. Annu. Rev. Cell Dev. Biol. 30, 357–391. 10.1146/annurev-cellbio-101011-155756 25288115

[B9] Opazo M. C. Ortega-Rocha E. M. Coronado-Arrázola I. Bonifaz L. C. Boudin H. Neunlist M.. (2018). Intestinal Microbiota Influences Non-intestinal Related Autoimmune Diseases. Front Microbiol. 9, 432. 10.3389/fmicb.2018.00432 29593681 PMC5857604

[B10] Sarkar R. S. Banerjee S. (2019). Gut microbiota in neurodegenerative disorders. J. Neuroimmunol. 15, 98–104. 10.1016/j.jneuroim.2019.01.004 30658292

[B11] Szendroedi J. Phielix E. Roden M. (2011). The role of mitochondria in insulin resistance and type 2 diabetes mellitus. Nat. Rev. Endocrinol. 8, 92–103. 10.1038/nrendo.2011.138 21912398

[B12] Yardeni T. Tanes C. E. Bittinger K. Mattei L. M. Schaefer P. M. Singh L. N.. (2019). Host mitochondria influence gut microbiome diversity: a role for ROS. Sci. Signal. 12, eaaw3159. 10.1126/scisignal.aaw3159 31266851

